# Nano hydrogel-based oxygen-releasing stem cell transplantation system for treating diabetic foot

**DOI:** 10.1186/s12951-023-01925-z

**Published:** 2023-06-27

**Authors:** Liangmiao Chen, Bingru Zheng, Yizhou Xu, Changzheng Sun, Wanrui Wu, Xiangpang Xie, Yu Zhu, Wei Cai, Suifang Lin, Ya Luo, Changsheng Shi

**Affiliations:** 1 Department of Endocrinology, The Third Affiliated Hospital of Wenzhou Medical University, 325200 Wenzhou, Zhejiang China; 2 Department of Interventional Vascular Surgery, The Third Affiliated Hospital of Wenzhou Medical University, No.108 Wansong Road, 325200 Wenzhou, Zhejiang China; 3 Institute of Cardiovascular Development and Translational Medicine, The Second Affiliated Hospital & Yuying Children’s Hospital of Wenzhou Medical University, 325027 Wenzhou, Zhejiang China

**Keywords:** Microspheres, Stem cells, Diabetic foot, Hydrogel, Transplantation

## Abstract

The employment of stem cells and hydrogel is widespread in contemporary clinical approaches to treating diabetic foot ulcers. However, the hypoxic conditions in the surrounding lesion tissue lead to a low stem cell survival rate following transplantation. This research introduces a novel hydrogel with superior oxygen permeability and biocompatibility, serving as a vehicle for developing a stem cell transplantation system incorporating oxygen-releasing microspheres and cardiosphere-derived stem cells (CDCs). By optimizing the peroxidase fixation quantity on the microsphere surface and the oxygen-releasing microsphere content within the transplantation system, intracellular oxygen levels were assessed using electron paramagnetic resonance (EPR) under simulated low-oxygen conditions in vitro. The expression of vascularization and repair-related indexes were evaluated via RT-PCR and ELISA. The microspheres were found to continuously release oxygen for three weeks within the transplantation system, promoting growth factor expression to maintain intracellular oxygen levels and support the survival and proliferation of CDCs. Moreover, the effect of this stem cell transplantation system on wound healing in a diabetic foot mice model was examined through an in vivo animal experiment. The oxygen-releasing microspheres within the transplantation system preserved the intracellular oxygen levels of CDCs in the hypoxic environment of injured tissues. By inhibiting the expression of inflammatory factors and stimulating the upregulation of pertinent growth factors, it improved the vascularization of ulcer tissue on the mice’s back and expedited the healing of the wound site. Overall, the stem cell transplantation system in this study, based on hydrogels containing CDCs and oxygen-releasing microspheres, offers a promising strategy for the clinical implementation of localized stem cell delivery to improve diabetic foot wound healing.

## Introduction

Diabetic foot, a severe diabetes mellitus complication, is a highly prevalent and hazardous condition that contributes to the primary source of disability and mortality among diabetic patients [1]. Evidence suggests that 4–10% of patients with type 2 diabetes develop diabetic foot ulcers, and the risk of death within five years is 2.5 times higher in patients with diabetic foot than in those with diabetes alone [2]. Additionally, amputation resulting from diabetic foot comprises 40-80% of non-traumatic amputation cases, with a mortality rate of up to 40% five years post-amputation [3]. Traditional diabetic foot treatments predominantly involve medications, which are both ineffective and costly. Moreover, single interventional or vascular surgery bypass treatments are insufficient, as they do not address the fundamental pathological basis of diffuse vascular stenosis and occlusion, as well as nerve and tissue breakdown in the diabetic foot. Consequently, there is an urgent clinical need for the discovery of new efficacious treatment methods.

In recent years, as stem cell research has advanced, stem cell-based therapy has emerged as a promising approach for treating diabetic foot and improving patient prognosis. Stem cells exhibit remarkable self-renewal capabilities and multi-directional differentiation potential, secreting a variety of cytokines. Stem cells employed in preclinical and clinical research include adipose mesenchymal stem cells, umbilical cord blood mesenchymal stem cells, and bone marrow stem cells, among others. Notably, bone marrow mesenchymal stem cells (MSCs) have demonstrated significant efficacy in treating diabetic neuropathy, as local MSCs transplantation can promote angiogenesis around the diabetic foot and increase neurotrophic factor production [4]. Moreover, autologous bone marrow stem cell transplantation has proven more effective in promoting nerve conduction in the affected limb, treating chronic lower limb ischemia, and improving patient prognosis than conventional treatment [5]. Furthermore, intravenously transplanted human umbilical cord mesenchymal stem cells (Hus-MSCs) have shown the ability to migrate and localize to wound tissue, expediting wound healing in a rat diabetic foot ulcer (DFU) model by modulating inflammation and promoting growth factor deposition associated with angiogenesis, cell proliferation, and collagen [6]. Despite stem cell therapy’s success in numerous basic studies conducted both domestically and internationally, encompassing in vitro and in vivo animal experiments, the retention and survival rates of transplanted stem cells in peripheral tissues remain exceedingly low, significantly limiting stem cell therapy’s efficacy and potential for clinical applications [7, 8]. Consequently, enhancing the retention and survival of transplanted stem cells is currently a crucial scientific issue in the realm of stem cell transplantation therapy.

Recent advancements in material science have led to the development of innovative materials with potential applications in biomedical engineering [9, 10]. Hydrogels are a novel type of functional polymer material characterized by a three-dimensional network structure, garnering widespread attention in the medical wound healing field due to their exceptional biocompatibility and functional properties [11]. Feng et al. [12] reported an injectable hydrogel prepared by heating a mixture of chitosan and graphene oxide solution, demonstrating significant potential in hemostasis and full-thickness skin wound repair. Additionally, injectable hydrogels, such as fibrin hydrogels, chitosan hydrogels, hyaluronic acid hydrogels, and polyethylene glycol hydrogels, are highly popular delivery vehicles. For instance, DivBand et al. [13] developed injectable hydrogels based on chitosan biguanide and hydroxymethyl cellulose, capable of forming in situ and loaded with VEGF and BMP-2 for studying the osteogenic differentiation of dental pulp stem cells. They discovered that these hydrogels possess the potential to serve as injectable platforms for bone tissue engineering. Despite this, currently, few specialized carriers are applied for stem cell transplantation. A possible strategy to address the issue of low stem cell implantation retention involves suspending the cells in a hydrogel matrix capable of providing a suitable microenvironment [14]. However, existing hydrogels lack the properties of an ideal cell carrier, which slow solidification rate may result in cell extrusion during muscle contraction and can often solidify in the bloodstream, causing additional tissue damage by obstructing blood vessels in the lower extremities. Therefore, it is crucial to develop a hydrogel that can serve as an ideal carrier to effectively address the issue of low retention rates of stem cells in implanted tissues.

Oxygen is a vital component of biological processes, and an oxygen-containing microenvironment during angiogenesis improves wound healing and promotes the epithelialization process. The majority of diabetic foot wounds are chronic, and characterized by hypoxia. Cell survival is limited in the ischemic limb’s hypoxic environment[15], leading to a very low implantation survival rate in stem cell therapy for the diabetic foot. Chronic hypoxia caused by disrupted vascular supply and chronic inflammation, impedes the wound healing process, making it essential to improve wound tissue oxygenation to support the repair of damaged tissue in the diabetic foot. Therefore, to truly realize the clinical application of stem cell therapy in the diabetic foot, it is necessary to create a microenvironment that significantly improves stem cell survival rate. Although common hypoxic preconditioning [16] and hyperbaric oxygen preconditioning [17] can somewhat enhance cell survival, the effect is relatively unspecific. Additionally, some studies have developed oxygen-release systems for local implantation to improve cell survival by ameliorating the hypoxic microenvironment at the cell implantation site, thereby better harnessing the therapeutic effects of stem cells. However, these oxygenation systems typically release oxygen in less than two weeks [18–21], while vascular growth, granulation, and re-epithelialization require at least three weeks, highlighting the critical need to develop oxygen-release systems that can provide oxygen to damaged tissues for extended periods. Previous studies have shown that the introduction of polylactic acid (PLA) during the preparation of oxygen-releasing microspheres has potential antibacterial properties. Composite materials synthesized with PLA can inhibit Escherichia coli, Staphylococcus aureus, and other bacterial strains [22–24]. Antibacterial PLA materials can extend the shelf life of food, reduce food spoilage, and waste. Additionally, PLA materials have been approved by the FDA for use in food packaging and medical devices, which means they can come into direct contact with the human body or food without any negative impact on human health.

In summary, we have developed a stem cell transplantation system based on novel hydrogels and oxygen-releasing microspheres that aim to address the two key challenges of stem cell transplantation for diabetic foot treatment, while also possessing the capability of releasing oxygen for more than 3 weeks. We hypothesize that this transplantation system can significantly improve the retention and survival rates of stem cells in the diabetic foot target tissue, thereby enhancing stem cells’ therapeutic effect on diabetic foot disease. Moreover, this study offers valuable insights into the clinical application of stem cells in diabetic foot treatment. To investigate the intracellular oxygen content, survival rate, and paracrine effects of GFP-positive cardiosphere-derived stem cells (CDCs) under hypoxic conditions induced by vascular and nerve tissue lesions in the distal affected limb of diabetic foot, we incorporated these cells into the transplantation system and conducted in vitro experiments. Additionally, we examined the therapeutic effects of CDCs on a diabetic foot animal model and their mechanism.

## Materials and methods

### Materials

Polyvinylpyrrolidone (PVP) with a molecular weight of 40 kDa, hydrogen peroxide (H_2_O_2_), catalase, sodium alginate, and poloxamer 407 were purchased from Sigma-Aldrich Corporation. Poly(lactide-co-glycolide)-b-Poly(ethylene glycol)-biotin (PLGA-PEG-Biotin) was procured from Xi’an Ruixi Biological Technology Co.,Ltd.

### Preparation of sodium alginate/poloxamer injectable temperature-sensitive hydrogel

In this study, a class of temperature- and pH-sensitive hydrogels were successfully fabricated. Initially, 1.2 g of sodium alginate powder and 1.2 g of poloxamer 407 powder were weighed into a beaker in a specific mass ratio of 1:1. Subsequently, 20 mL of ultrapure water was added to the beaker, thoroughly mixed using a magnetic stirrer set at 400 rpm for 30 min at room temperature, and then the beaker was transferred to a refrigerator at 4 ℃ for 24 h to ensure complete dissolution. This process ultimately yielded injectable temperature-sensitive composite hydrogel samples. The concentrations of sodium alginate and poloxamer 407 in the gel solution were both 6%.

### Characterization of hydrogel properties and morphology

The molar ratio of each component of the hydrogel was adjusted to determine the gelation temperature of the hydrogel at different pH conditions, the rigidity, elongation at break, and degradation of the hydrogel under characteristic conditions to study the properties of the hydrogel.

Oxygen permeability is vital for cell growth in hydrogels. To assess the oxygen permeability of the hydrogels, the oxygen partial pressure (PO_2_) at 21% O_2_ was measured using electron paramagnetic resonance (EPR) with oxygen-sensitive lithium phthalocyanine (LiPc) as the EPR probe. Additionally, CDCs with a density of 2.5 × 10^6^/mL were embedded in the hydrogel and cultured at room temperature under normal conditions of 21% O_2_ for one week. The content of double-stranded DNA (dsDNA) in living cells was utilized to reflect the survival and growth of cells, thereby characterizing the biocompatibility of the hydrogel. The dsDNA will be quantified using the PicoGreen dsDNA assay kit.

To further characterize the hydrogel morphology, the hydrogel samples were subjected to nanoparticle tracking analysis (NTA) and scanning electron microscopy (SEM). Nanosight NS300 (Malvern Panalytical, UK) was employed for NTA to determine the size distribution and concentration of nanoparticles in the hydrogels. Samples were diluted with ultrapure 1× PBS at a ratio of 1:1000 and then injected into the NS300 machine using a 1 mL syringe. The laser beam was passed through the sample chamber, and the particles were visualized by a microscope equipped with a camera to capture the Brownian motion of the nanoparticles and calculate the concentration and hydrodynamic diameter based on their motion using Einstein’s equation. The prepared hydrogels were freeze-dried and the microscopic morphology of the freeze-dried gels with gold sprayed on the surface was observed using SU8010 field emission SEM (HITACHI, Japan).

### Preparation of oxygen release system

**Fig. 1:**
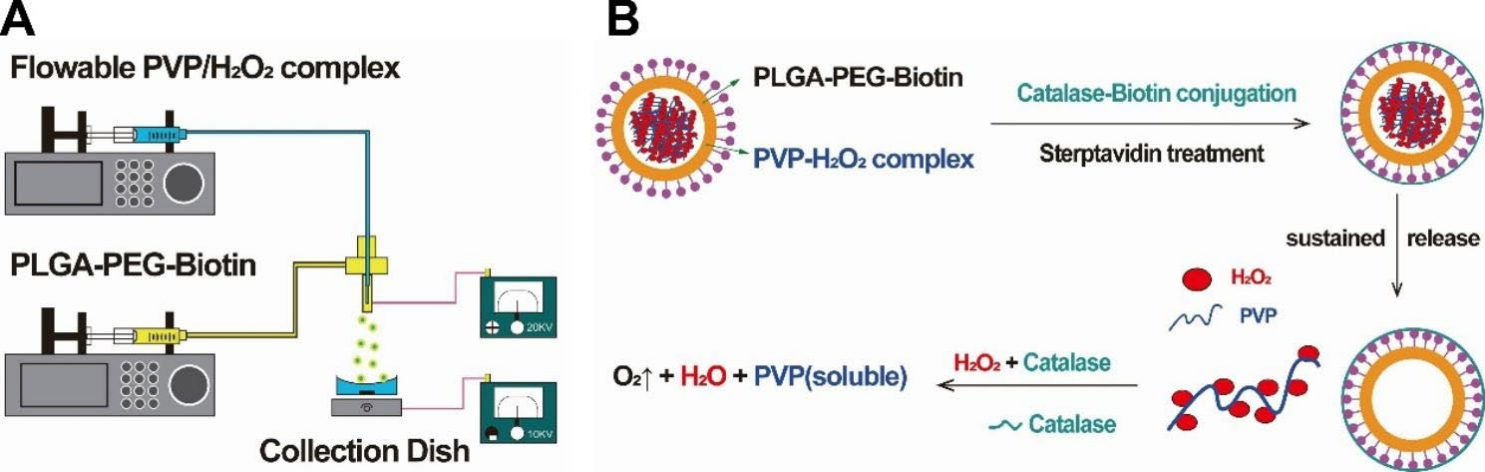
Preparation of oxygen release system. **A** Schematic representation of core-shell structured microspheres produced by the co-axial electrospraying technique. **B** Schematic illustration of the PLGA shell of oxygen-releasing microspheres with surface-immobilized peroxidase constructed with PLA-PEG-Biotin.

The oxygen release system comprises PVP/H_2_O_2_-releasing core-shell structured microspheres and peroxidase. The oxygen-releasing microspheres were prepared to utilize the well-established electrospray technique [25, 26], employing a coaxial stream and a collection dish at + 20 kV and − 10 kV, respectively (Fig. 1A). The microspheres featured a core and a shell composed of PVP/H_2_O_2_ and PLGA, respectively. PVP/H_2_O_2_ was synthesized by dissolving a specific quantity of PVP in a hydrogen peroxide solution. The PLGA-PEG-Biotin solution was created by dissolving PLGA-PEG-Biotin in dichloromethane achieving a concentration of 10%. At this concentration, the PLGA-PEG-Biotin solution exclusively generated microspheres and no fibers post-electrospraying. In this research, we immobilized catalase on the core-shell microspheres’ surface to establish the oxygen release system, facilitating the conversion of H_2_O_2_ to oxygen. Biotin in the copolymer was employed to immobilize biotinylated catalase following anti-biotin protein treatment. Incorporating polyethylene glycol (PEG) into the copolymer assisted in dispersing biotin within the aqueous medium, streamlining the immobilization process (Fig. 1B). Consequently, surface-immobilized hydrogen peroxidase shells were synthesized, ensuring the successful immobilization of hydrogen peroxidase on the microspheres’ exterior surface. Thus, PVP/H_2_O_2_ is progressively released during PLGA degradation, later transforming into oxygen through catalase.

### Optimization of catalase concentration immobilized on microsphere surface

When utilizing oxygen-releasing microspheres, surface-immobilized catalase should rapidly and thoroughly convert the PVP/H_2_O_2_ released internally following PLGA degradation to oxygen, preventing cellular damage caused by direct contact between H_2_O_2_ and cells. Prior research has determined that incorporating 1 mg/mL catalase-immobilized microspheres into the hydrogel can swiftly convert the released H_2_O_2_ into oxygen within two weeks. Consequently, for subsequent investigations, varying catalase concentrations (1, 2, 4 mg/mL) will be immobilized on the microspheres’ surface, different microspheres will be integrated into the hydrogel, and 1 × 10^7^/mL GFP-positive rat CDCs will be included. After curing the hydrogels at 37 ℃ and incubating them in 1% O_2_ and 1% PBS for 1, 3, 7, 10, 14, 18, and 21 days, the H_2_O_2_ content in the culture solution will be quantified using the Amplex Red hydrogen peroxide/peroxidase assay kit. The double-stranded DNA (dsDNA) quantity in living cells will be assessed using the PicoGreen dsDNA assay kit. The results will establish the optimal peroxidase immobilization concentration for subsequent studies.

### Characterization of oxygen release kinetics

Oxygen release kinetics are contingent upon the prompt conversion of the released PVP/H_2_O_2_ from PLGA by catalase immobilized on the surface of the microspheres, thereby ensuring a continuous oxygen supply. To develop a stem cell transplantation system capable of sustained oxygen release under hypoxic conditions for three weeks, as required in this study, determining the appropriate oxygen-releasing microsphere content is essential. Insufficient or excessive microsphere content may hinder the effective provision of a continuous oxygen supply over a three-week period. Accordingly, five different microsphere content (2.5, 5, 10, 15, and 20 wt%) were incorporated into the synthetic hydrogel carriers mentioned earlier, along with 1 × 10^7^/mL rat CDCs. After 1, 7, 14, and 21 days of hypoxic incubation, oxygen release kinetics were characterized by measuring intracellular oxygen content using EPR.

### Characterization of cell transplantation systems to enhance cellular oxygenation, survival, and paracrine effects under hypoxic conditions

Intracellular oxygen content directly influences cell survival. Consequently, in the oxygen release kinetic characterization experiments mentioned above, hydrogels containing CDCs and oxygen-releasing microspheres were cured at 37 ℃ and incubated in 1% O_2_ and 1% PBS for 1, 7, 14, and 21 days. In addition to measuring intracellular oxygen content, cell survival was assessed by quantifying cellular dsDNA content to comprehend the cells’ utilization of released oxygen to improve their survival. Furthermore, stem cell therapeutic mechanisms have been closely linked to paracrine effects. Thus, the experiment investigated the impact of oxygen release on CDCs paracrine effects to confirm that oxygen released from stem cells could enhance paracrine action, thereby playing a more effective role in diabetic foot stem cell therapy and achieving the anticipated therapeutic outcome. A hydrogel solution was employed to encapsulate oxygen-releasing microspheres and CDCs as the + O_2_ release group, while a gel embedding CDCs without oxygen-releasing microspheres served as the control. The samples were cultured for 21 days in 1% O_2_ and 1% PBS.

### Establishment of diabetic foot animal model

Female db/db mice (6–8 weeks) were obtained from the Institute of Model Animal Research, Wenzhou University. The mice had unrestricted access to standard food and water and were subjected to a 12-hour light/dark cycle at a constant temperature and humidity. To establish a diabetic foot ulcer mouse model, the mice were restrained, and the skin at the midline of the spine, 2 cm below the scapula level on the mice’s backs, was selected as the modeling area. After disinfection, a specialized skin puncture device was employed to generate a 6–8 mm skin injury wound in the modeling region, and the full-thickness skin (extending to the fascia) was excised. All animal protocols were approved by the Wenzhou Medical University Laboratory Animal Center (ethical numbers: wydw 2019 − 0886).

### Evaluation of stem cell transplantation system’s effect on ulcerated skin in diabetic foot mice

After successfully constructing the diabetic foot skin ulcer model, animals were divided into the following groups: A: no treatment (MI), B: injection of hydrogel only (Gel), C: injection of hydrogel encapsulated with CDCs (Gel/Cell), D: injection of hydrogel encapsulated with oxygen-releasing microspheres and CDCs (Gel/O_2_), with at least six animals in each group. Thirty minutes after model construction, hydrogels under different loading conditions were directly injected intramuscularly into the skin wounds of diabetic foot model mice at a dosage of 200µL/mouse.

To study the visible effect of the stem cell transplantation system on diabetic foot mice, we photographed and documented the wounds on the mice’s back at 0, 3, 7, and 14 days post-transplantation and analyzed the healing of the damaged skin. Additionally, we measured the intracellular oxygen content of CDCs transplanted into the body to investigate whether the oxygen-releasing microspheres could maintain the same performance of continuous oxygen release for three weeks in vivo. The measurement was conducted using a non-invasive assay without requiring surgery on the animal.

### Enzyme-linked immunosorbent assay

The protein content of CDCs was determined using the MicroBCA assay for the in vitro culture experiment. The culture medium was collected, and the secretion levels of bFGF, HGF-1, VEGF, PDGF, and ANGPT1 were assessed using an ELISA kit (R&D Systems, Minneapolis, MN, USA). The ELISA kit employed a double antibody sandwich method, wherein specific monoclonal antibodies were coated, and enzyme-linked specific polyclonal antibodies were used as secondary antibodies for detection. The absorbance of the samples was measured at 450 nm, and the protein concentration was calculated according to the standard curve, following the manufacturer’s instructions.

### Fluorescent quantitative PCR assay

In this study, the mRNA expression levels of bFGF, HGF-1, VEGF, PDGF, and ANGPT1 in CDCs under hypoxic and aerobic conditions were detected by RT-PCR. Additionally, the mRNA expression levels of IL-6, α-SMA, CD31, Collagen I, Collagen III, CD80, CD206, and Arg-1 were measured in vivo by RT-PCR. The effects of oxygen release on paracrine action and the expression of related factors in vascularization and tissue repair of mouse ulcer skin were studied by examining the corresponding indicators’ expression. The primer gene sequences were designed by NCBI itself, and the sequence information is displayed in Table 1, with no significant homology found after an NCBI BLAST search.

**Table 1:** List of Primer Sequences for Real-Time PCR

Primer name	Primer sequence (5’→3’)
GAPDH-F	GGAGCGAGATCCCTCCAAAAT
GAPDH-R	GGCTGTTGTCATACTTCTCATGG
bFGF-F	GCAGAAGAGAGAGGAGTTGT
bFGF-R	ACCGGTAAGTATTGTAGTTA
IGF-1-F	TATGGCTCCAGCAGTCGGAGG
IGF-1-R	TGGGTCTTGGGCATGTCGGT
HGF-F	ATGATGTCCACGGAAGAGGA
HGF-R	TTGTGCATCCATAATTAGGT
VEGF-F	TACCCTGATGAGATCGAGT
VEGF-R	CTCCTATGTGCTGGCCTTGGT
PDGF -F	AGATTCCTCGGAGTCAGGTCG
PDGF-R	ACGTATTCCACCTTGGCCAC
ANGPT1 -F	TATTCACAGTATGACAGATT
ANGPT1-R	GCACATTTGCACATACAGT
IL-6-F	CCACTTCACAAGTCGGAGGCTTA
IL-6-R	GCAAGTGCATCATCGTTGTTCATAC
α-SMA-F	TGACCCAGATTATGTTTGAGACC
α-SMA-R	CCAGAGTCCAGCACAATACCA
CD31-F	TCATTGCGGTGGTTGTCATT
CD31-R	TTGGCTTCCACACTAGGCTCAG
Collagen I-F	AGGCCACGCATGAGCCGAAG
Collagen I-R	GCCATGCGTCAGGAGGCAG
Collagen III -F	AGGATCTGAGGGCTCGCCAGG
Collagen III -R	AGCCACCAGACTTTTCACCTCCA
CD80-F	TATGGAGATGCTCACGTGT
CD80-R	TTCTGTGCTTACAGAAGCA
CD206-F	CGAGACTGCTGCTGAGTCC
CD206-R	GTACGAAGAACAGTGGATA
Arg-1-F	TCTCTACATCACAGAAG
Arg-1-R	GTTCACAGTACTCTTCA

### Histological and immunochemical staining analysis

Three weeks after injecting the stem cell transplantation system, full-thickness skin (1.5 × 1.5 cm) from the wound was taken, fixed with paraformaldehyde solution, embedded in paraffin, and sectioned for hematoxylin/eosin staining. The remaining sections were stained with CD34 and α-SMA immunohistochemistry. The staining results were analyzed after capturing microscopic images.

### Statistical analysis

Graph Pad Prism 8.0 software was used for statistical analysis, with all data expressed as mean ± standard deviation. One-way ANOVA was used for comparing multiple groups, and statistical significance was defined as *P* < 0.05.

## Results

### Temperature and pH-sensitive hydrogels are suitable as carriers for stem cell transplantation

**Fig. 2:**
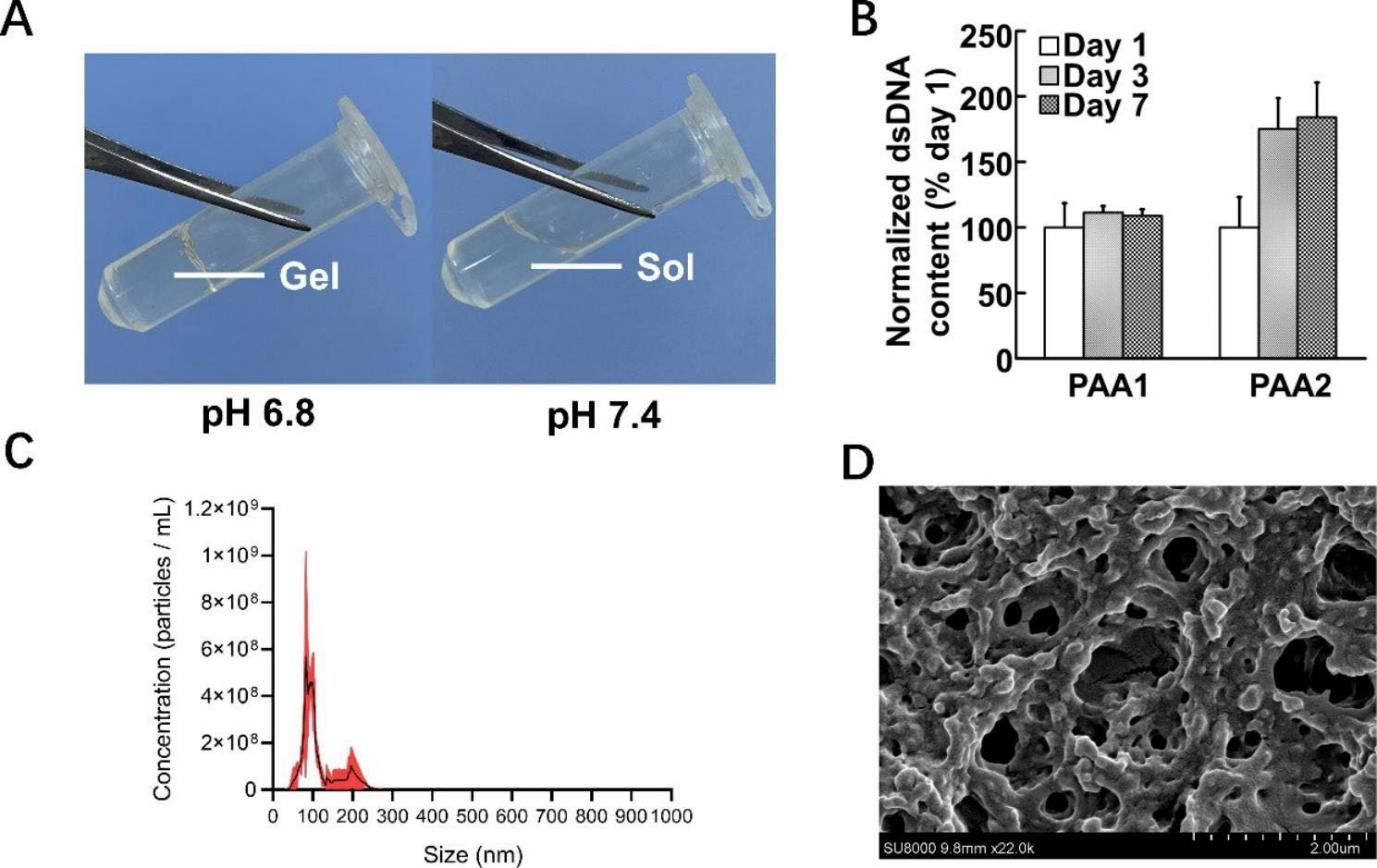
Temperature- and pH-sensitive hydrogels are suitable as stem cell transplantation vectors. **A** Injectability of hydrogels with gel-forming properties at different pH. **B** Survival growth of CDCs in hydrogels in normal culture for one week (n = 3). **C** Size distribution of particles using NTA. **D** SEM images of hydrogels

The stem cell transplantation system is based on fast-gel hydrogel and oxygen-releasing microspheres. Figure 2A; Table 2 demonstrate that the pH 7.4 hydrogel solution can rapidly solidify (< 10s) in the pH 6.8 buffer at 37℃ due to the sol-gel temperature being lower than 37 ℃ at this pH condition (Table 2). Simultaneously, these hydrogel solutions cannot solidify in a 37 ℃, pH 7.4 buffer solution (Fig. 2A) because their sol-gel temperature is greater than 37℃ under this condition, so these hydrogel solutions cannot solidify in the blood. All solutions can be injected through a 28G interventional catheter at 37℃. As shown in Table 2, hydrogels with molar ratios of 78/13/9 versus 72/13/15 for each component are more suitable as transplantation carriers for stem cells in this study due to lower stiffness, and greater weight loss after eight weeks at 37 ℃ in pH 6.8 buffer indicating better degradation properties.

**Table 2:** Modulating hydrogel composition to tailor hydrogel gelation temperature at different pH, mechanical and degradation properties

Polymer	Composition^*^ (mol%)	Gelation Temp (℃)	Stiffness (kPa)	Tensile strain^**^ (%)	Weight loss^***^ (%)
PAA1	72/13/15	33.5 (pH 6.8)	36.5 ± 6.9	> 300	15.7 ± 3.0
		39.6 (pH 7.4)			
PAA2	78/13/9	35.7 (pH 6.8)	5.4 ± 1.9	> 300	36.1 ± 3.5
		41.3 (pH 7.4)			
PAA3	77/8/15	30.3 (pH 6.8)	52.6 ± 2.3	> 300	5.8 ± 1.6
		39.3 (pH 7.4)			

^*^NIPPAm/PAA/macromer (HEMAPTMC) ratio. The number of TMC unites in HEMAPTMC is 2; ^**^Out of strain range of the testing equipment. ^***^ after 8 weeks in PH 6.8 buffer at 37 ℃

The oxygen permeability of hydrogels is essential for cell growth in hydrogels. The hydrogel’s oxygen permeability was detected by EPR and an oxygen detection probe, and the oxygen partial pressure (157.2 mmHg) in the hydrogel was similar to that in PBS (158.6 mmHg). The hydrogel’s superior oxygen permeability property can effectively increase cell survival under oxygen-release conditions. Additionally, when CDCs with a density of 2.5 × 10^6^/mL were embedded into the hydrogel, the hydrogel remained highly elastic with an elongation at a break of > 300%. After seven days of incubation under normal conditions, the dsDNA results (Fig. 2B) indicated that CDCs could survive or proliferate in the hydrogel. These results suggest that the hydrogel synthesized in this study was suitable as a carrier for CDCs to be transplanted into the surrounding tissue of the necrotic diabetic foot.

The NTA peak profile showed an average nanoparticle size of 93.8 nm for the hydrogel (Fig. 2C). Examination of the morphology of the freeze-dried hydrogel via SEM revealed a distinct internal porous structure, forming a three-dimensional network-like configuration. No apparent layering between the materials was observed, indicating good compatibility among the raw materials (Fig. 2D).

### Preparation of oxygen-releasing microspheres with surface immobilized hydrogen peroxidase

**Fig. 3:**
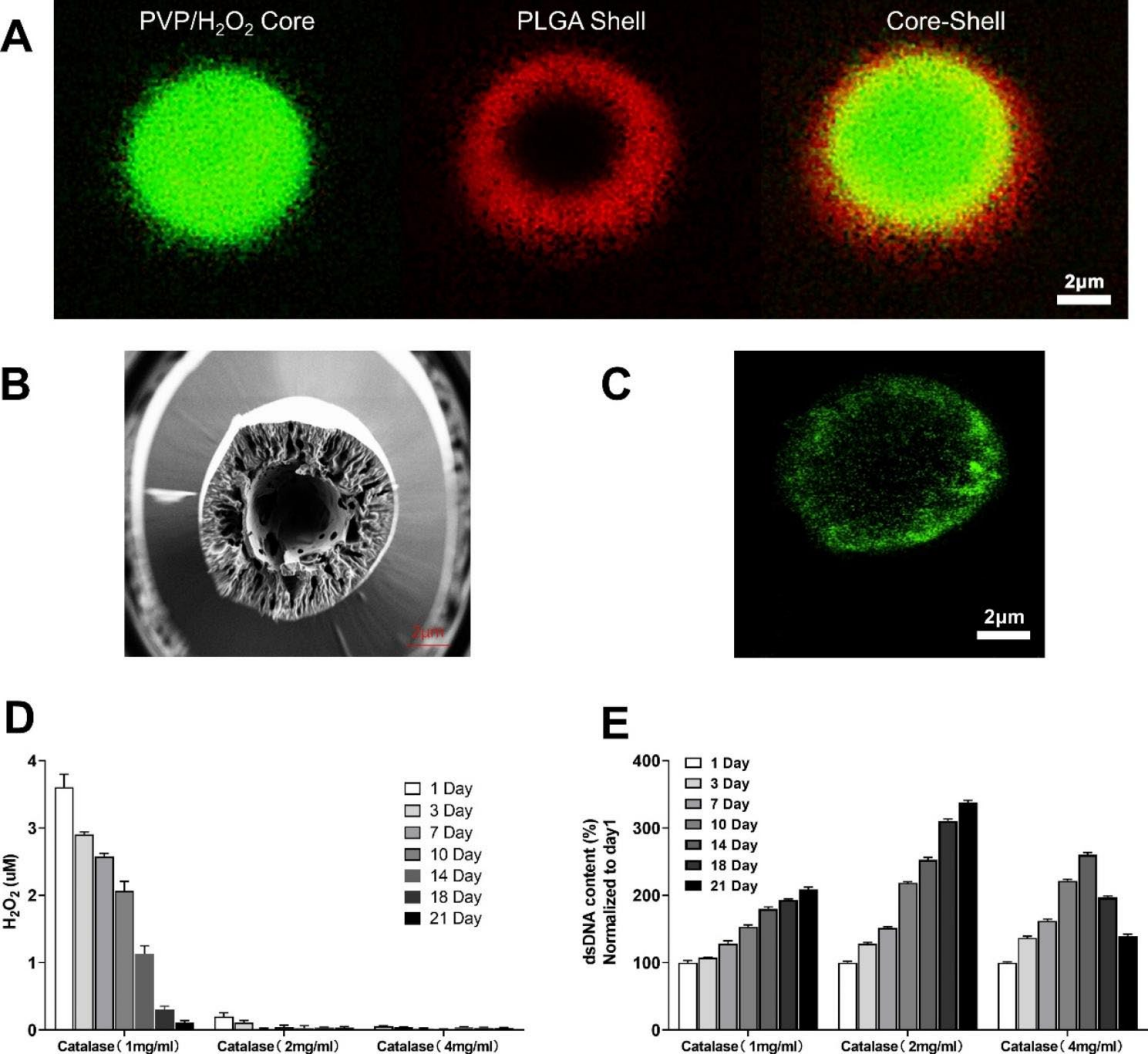
Preparation of surface-immobilized catalase-immobilized oxygen-releasing microspheres. **A** The oxygen-releasing microspheres have a core-shell structure, with PVP/H_2_O_2_ as the core and PLGA as the shell. **B** Scanning electron microscope image of core-shell microspheres. **C** Confocal microscopy image of catalase-immobilized oxygen-releasing microspheres. **D** Changes of hydrogen peroxide content in the culture medium within three weeks after the stem cell transplantation system with different concentrations of catalase-immobilized microspheres were solidified at 37℃ in 1% O_2_ and 1% PBS (n = 3). **E** The living cells of the stem cell transplantation system containing different concentrations of catalase-immobilized microspheres were solidified at 37℃ and cultured in 1% O_2_ and 1% PBS for three weeks (n = 3)

The oxygen-releasing microspheres were prepared by the electrospray method and had a core-shell structure with PLGA as the shell and PVP/H_2_O_2_ complex as the core (Fig. 3A, B). Additionally, catalase was immobilized on the surface of PLGA microspheres by adding block copolymers in PLGA by the above method. The prepared microspheres were dispersed in PBS, and then streptavidin protein solution was added. After being washed with PBS, the microspheres were added to a biotinylated fluorescein isothiocyanate-labeled catalase solution. Finally, the ideal surface immobilized catalase oxygen-release microspheres were obtained (Fig. 3C).

Inappropriate concentrations of catalase in the experiment could affect cell survival over a three-week period. The concentration of catalase was optimized for this experiment based on the following criteria: it would not cause dramatic cell death at any time point during the three weeks, and no H_2_O_2_ would be detectable in the culture medium at any time point. As shown in Fig. 3D, effective decomposition of H_2_O_2_ in the culture medium was achieved when the fixed concentration of catalase was 2 and 4 mg/mL, ensuring that there was no excessive accumulation of H_2_O_2_ to affect the normal survival of CDC. Additionally, the results of dsDNA content in CDC live cells (Fig. 3E) showed a certain increase in dsDNA content within three weeks when the peroxidase concentration was 1 and 2 mg/mL. Notably, a significant increase in dsDNA content was observed in the system with a peroxidase concentration of 2 mg/mL for three weeks, indicating the proliferation of CDC live cells. However, when the concentration was increased to 4 mg/mL, the peroxidase level was too large, resulting in premature completion of H_2_O_2_ decomposition and thus acute cell death due to hypoxia from day 14 onwards. Summing up the two results above, it can be concluded that 2 mg/mL is the best peroxidase concentration in this study that can completely convert H_2_O_2_ into oxygen in the nucleon-shell microspheres in time to support the effect of sustained oxygen release over 3 weeks.

### Stem cell transplantation system promotes cellular oxygenation, survival and paracrine effects under hypoxic conditions

**Fig. 4:**
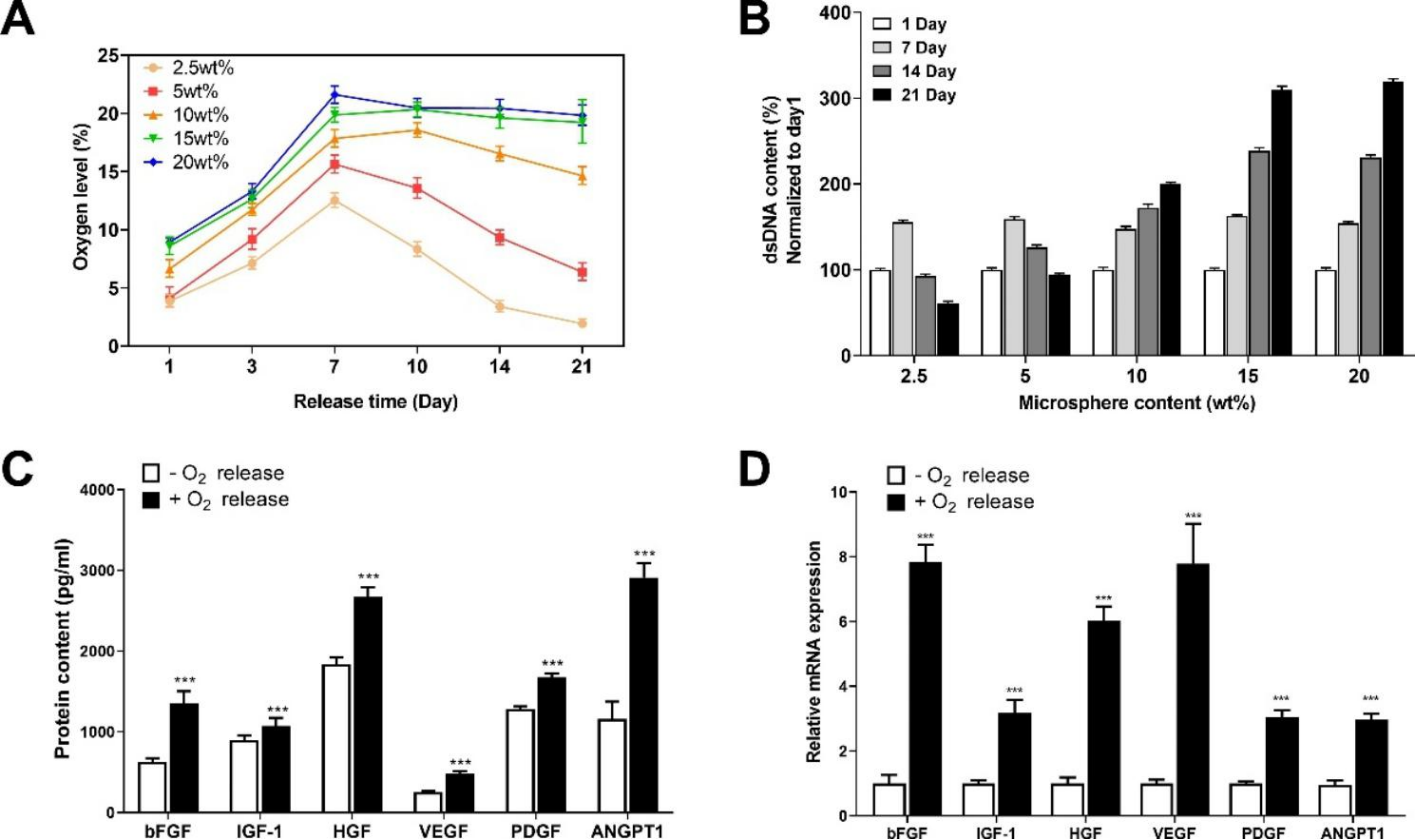
Effects of stem cell transplantation system on cellular oxygenation, survival, and paracrine effects under hypoxic conditions. **A** Oxygen release kinetics in stem cell transplantation systems with different microsphere contents within three weeks (n = 3). **B** The situation of viable cells within three weeks of culture in stem cell transplantation system with different microsphere content (n = 3). **C** Changes of paracrine-related growth factor protein content with or without oxygen release after 21 days of culture under 1% oxygen condition (n = 3). **D** Changes in paracrine-related growth factor mRNA expression levels with or without oxygen release after 21 days of culture under 1% oxygen condition (n = 3). ***P < 0.001

The content of oxygen-releasing microspheres affects the oxygen content in CDC cells, which in turn affects their survival. The three-week oxygen release kinetics of the five transplantation systems with different microsphere contents are shown in Fig. 4A. It is apparent that the intracellular oxygen content assay of CDC showed that the transplantation systems with 2.5, 5, and 10 wt% microsphere content were not effective in providing continuous oxygen for three weeks. In contrast, the transplantation systems with 15 and 20 wt% microspheres were able to release oxygen steadily for three weeks, maintaining the oxygen content in the cells at about 20%, which is beneficial for cell growth. In addition, the cell survival of different microsphere content groups was shown in Fig. 4B. It was found that when the microsphere content was too low, such as 2.5 and 5 wt% groups, the transplantation system could not generate enough oxygen to support cell survival within three weeks. Therefore, from the first week, the cell dsDNA content decreased significantly, indicating the rapid death of living cells. However, when the microsphere content was increased to 10 wt% and above, it was clearly seen that the three groups of dsDNA content, 10, 15, and 20 wt%, continued to increase in the assay over three weeks, indicating that the transplant system provided sufficient oxygen content to support cell survival and growth, and thus the cells continued to grow. The faster cell growth in the 15 and 20 wt% groups indicated that the content of these two oxygen-releasing microspheres was better than 10wt% and had better biocompatibility in this study.

In addition, the effect of oxygen release on the paracrine effect of CDC was characterized by RT-PCR and ELISA results. As shown in Fig. 4C, D, compared with the hypoxic environment, in the aerobic environment, bFGF, IGF-1, HGF, VEGF, PDGF, and ANGPT1, which are growth factors closely related to cell survival and vascularization, showed a substantial increase in both gene expression by RT-PCR and protein concentration by ELISA, and the significant differences were statistically significant (*P* < 0.001). Thus, the oxygen released from the microspheres in the cell transplantation system effectively promoted cell survival by upregulating the expression of paracrine effect-related growth factors.

### Stem cell transplantation system promotes healing of back ulcer in diabetic foot model mice

**Fig. 5:**
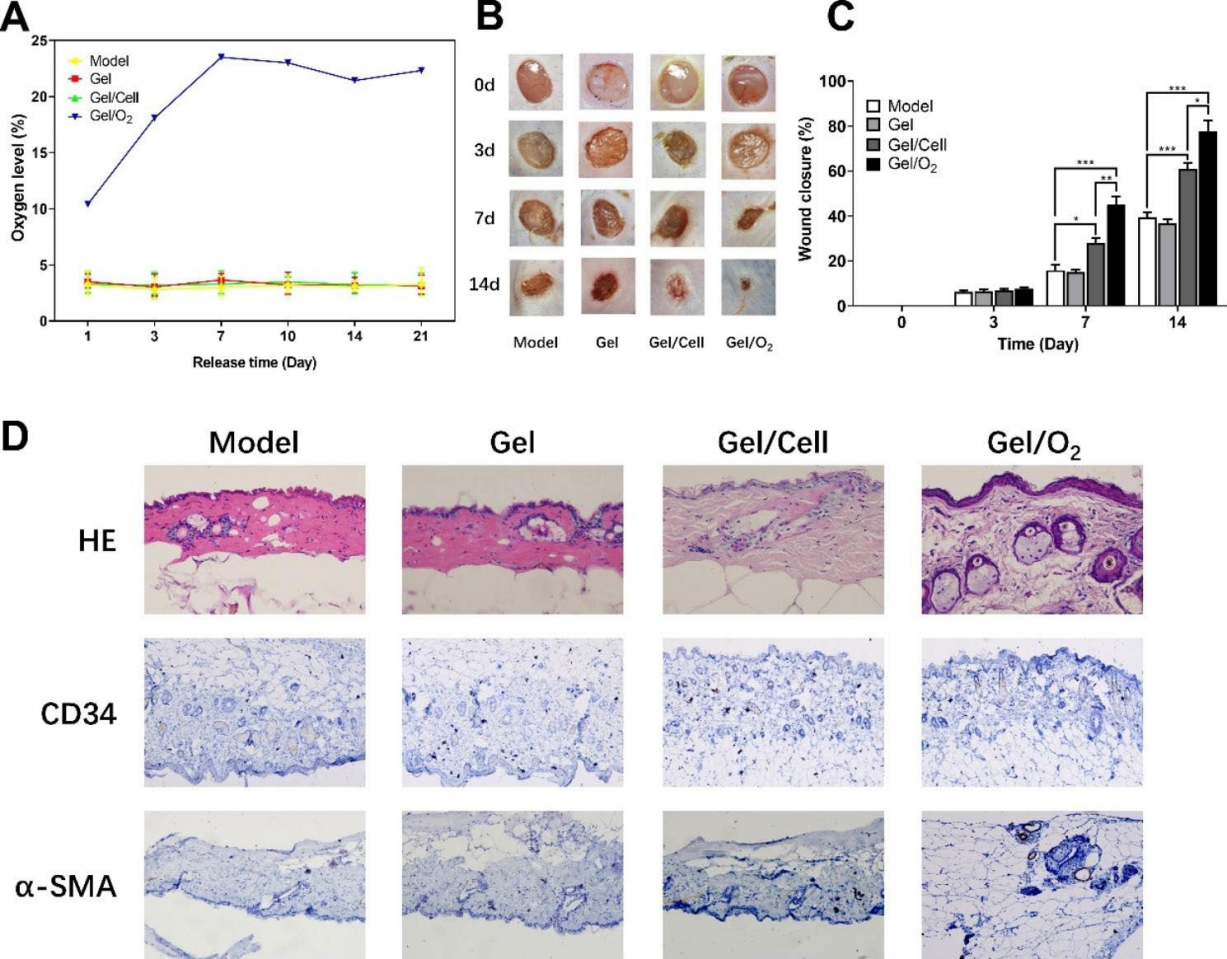
Effects of stem cell transplantation system on the healing of back ulcers in diabetic foot model mice. **A** The levels of intracellular oxygen content in different groups of CDCs within three weeks after transplantation. **B** The healing of the skin of the back ulcers of the diabetic foot model mice in different groups within two weeks. **C** The healing rate of ulcer wounds of mice in different groups within two weeks. *P < 0.05, **P < 0.01 and ***P < 0.001. **D** Three weeks after intramuscular injection of different systems into mouse back skin wounds, full-thickness skin samples were taken for HE staining and CD34 and α-SMA immunohistochemical staining

First, according to the results of the cellular oxygen content of CDC transplanted into the body for three weeks, only the group of transplantation system injected with oxygen-releasing microspheres was able to provide a continuous supply of oxygen for three weeks to maintain intracellular oxygen content of CDC suitable for survival (Fig. 5A), so this group would have a considerable post-transplantation survival rate and would have the best effect in wound healing in diabetic foot model mice.

In this experiment, a diabetic foot skin ulcer model was constructed by perforating the back of diabetic mice to evaluate the effect of the oxygen-releasing microspheres and hydrogel-based stem cell transplantation system on wound healing. The ulcer wound healing was recorded within 14 days (Fig. 5B), and the wound area was additionally measured for different groups at each time point to assess the wound healing. According to the results, it was found that only the injection gel group was close to the model control group, and the wound healing was slow, while the injection of only the hydrogel group loaded with stem cells and the hydrogel group loaded with oxygen release microspheres and stem cells could be found on the 7th day. The wound healing was significantly accelerated, and on the 14th day, the wound healing rate of the back of the two groups of mice reached about 62% and 78% (Fig. 5C), and the skin regeneration was smooth. Obviously, the significantly increased wound healing in the two groups compared to the model group demonstrated that the transplant systems injected in the two groups were effective.

The macroscopic results demonstrated that the transplantation system based on oxygen-releasing microspheres, stem cells, and hydrogels accelerated the contraction of diabetic foot wounds. To gain further insights, histological analysis was performed (Fig. 5D). The results of HE staining revealed that the groups injected with encapsulated stem cells hydrogel and injected with encapsulated stem cells and oxygen-releasing microspheres hydrogel exhibited a larger epithelial area and thicker granulation tissue growth compared to the model group and the group injected with hydrogel only. The latter showed more significant changes, indicating better re-epithelialization and tissue repair in the ulcerated skin of mice after treatment with the transplantation system. Additionally, the analysis of immunohistochemical results of CD34 and α-SMA demonstrated that the hydrogel group injected with encapsulated stem cells only had elevated expression of CD-34 with α-SMA compared to the model group and the hydrogel-only group. This suggests more blood vessel formation, indicating increased vascularization of skin tissue, which favors the repair of traumatized tissue. Moreover, the hydrogel group injected with encapsulated stem cells and oxygen-releasing microspheres exhibited higher CD34 and α-SMA expression, indicating more significant vascularization and tissue repair processes. This finding is consistent with the healing of ulcerated skin tissue detected phenotypically.

**Fig. 6:**
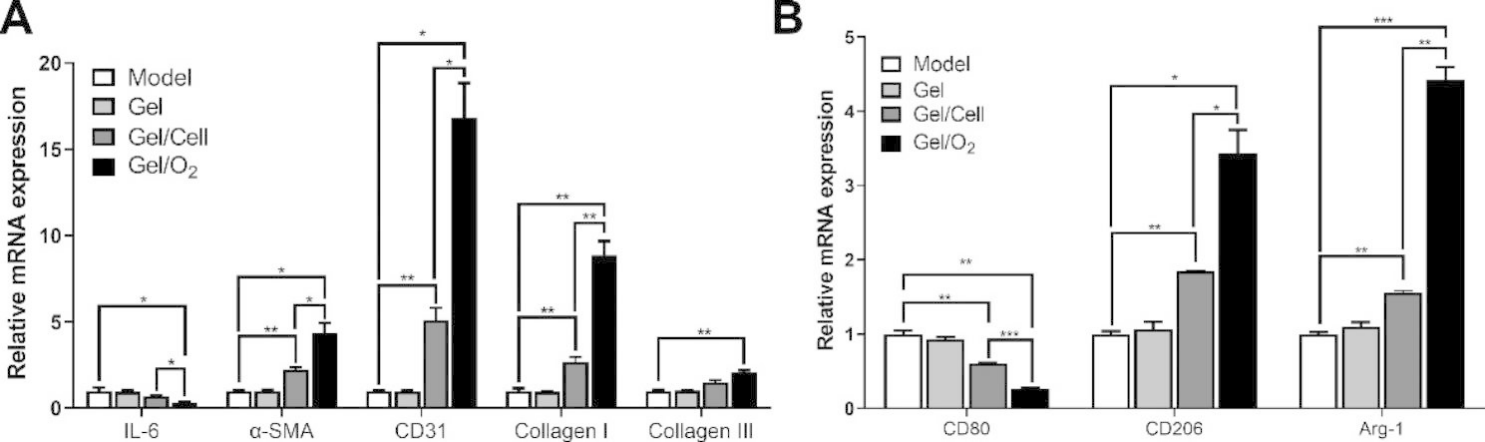
Effect of stem cell transplantation system on indices related to ulcer tissue repair and vascularization. Changes of mRNA expression levels of **A** vascularization and **B** repair-related indexes in ulcer tissue of diabetic foot model mice in different groups. *P < 0.05, **P < 0.01 and ***P < 0.001

The results of real-time quantitative fluorescence PCR indicators related to ulcer tissue repair and vascularization are shown in Fig. 6A, B. The results demonstrated that, among all the detection indicators, only the hydrogel injection group had similar expression levels to the model group. Compared with the model group and the hydrogel-injection-only group, the mRNA expression indexes of IL-6 and CD80 were significantly decreased in the stem cell-injection-only group, while the decrease was more significant in the hydrogel-injection group with stem cells and oxygen-releasing microspheres. This suggests that encapsulated stem cells and oxygen-releasing microspheres exerted some tissue inflammatory response inhibition, respectively, with the latter being more effective due to higher stem cell infusion survival rates. Furthermore, the significantly higher expression of α-SMA, Collagen I, Collagen III, and CD31 mRNA in the hydrogel group injected with encapsulated stem cells and the hydrogel group injected with encapsulated stem cells and oxygen-releasing microspheres compared with the model group and the hydrogel-only group indicated collagen deposition, endothelial cell growth, and contracture of scar tissue within the ulcerated skin. Moreover, high expression of CD206 and Arg-1 mRNA in both groups indicated polarization of type 2 macrophages, exerting anti-inflammatory effects and promoting wound repair and fibrous degeneration. Among them, again, the injection of encapsulated stem cells with oxygen-releasing microspheres hydrogel group had the best effect.

## Discussion

The application of topical stem cell transplantation for diabetic foot treatment has been extensively investigated. Stem cell therapy has demonstrated its potential to promote wound healing and ameliorate diabetic foot conditions by synthesizing and secreting cytokines that facilitate immune regulation, extracellular matrix remodeling, and angiogenesis [4, 27, 28]. However, the absence of suitable cell carriers and the hypoxic microenvironment surrounding the diabetic foot impede the survival rate of stem cells post-transplantation, thus limiting the comprehensive clinical implementation of stem cell therapy. In this study, we developed a stem cell transplantation system employing temperature- and pH-sensitive hydrogels and core-shell microspheres with sustained oxygen-releasing properties for three weeks. The introduction of this transplantation system resulted in improved engraftment survival rates for CDCs, consequently promoting enhanced diabetic foot ulcer wound healing in animal models. Additionally, we found that the system’s therapeutic mechanism is associated with paracrine signaling.

In our research, we successfully constructed a temperature- and pH-sensitive hydrogel primarily comprising NIPAAm, PAA, and a macromonomer based on HEMA and PTMC. NIPAAm serves as a degradable thermosensitive component, while PAA imparts pH sensitivity and elevates hydrogel water solubility [29, 30], and macromonomer contributes to degradability [31, 32]. Wound healing is a dynamic process accompanied by pH changes, and dressings with pH-responsive capabilities can selectively enhance local drug release in the acidic wound microenvironment during the inflammatory phase [33, 34]. Given the temperature and pH-sensitive attributes of this hydrogel, it exhibits rapid curing properties at the pH of diabetic foot necrotic tissue, but not at blood pH levels. The hydrogel demonstrates excellent biocompatibility and oxygen permeability, curing within 10 s at pH 6.8 and 37 ℃ without cross-linking agents, thereby allowing the swift fixation of encapsulated stem cells in diabetic foot ulcer wound tissue without compromising stem cell viability. Stem cell transplantation is currently employed extensively for chronic wound treatment and ischemic limb revascularization. However, the hypoxic microenvironment of damaged tissues results in low post-transplantation cell survival rates, and angiogenesis within damaged tissues requires at least three weeks [35]. Studies revealed that over 90% of MSCs die after 14 days of culture at 1% O_2_ [36], significantly impacting stem cell efficacy [35]. Consequently, providing oxygen to transplanted cells to disrupt the hypoxic environment becomes essential for enhancing cell survival rates. Furthermore, direct oxygen supplementation following cell transplantation has been proven to bolster cell survival [37, 38]. At present, common oxygenation techniques include hyperbaric oxygenation (HBO) [39], in situ tissue oxygen generation [40], and oxygen carriers [41–43].

In this study, we prepared oxygen-releasing microspheres with PVP/H_2_O_2_ and PLGA as core-shell components, immobilizing hydrogen peroxidase on the outer surface to promptly convert H_2_O_2_ released during PLGA degradation, into oxygen. By optimizing hydrogen peroxidase immobilization and microsphere content within the transplantation system, we achieved oxygen release performance for three weeks, maintaining oxygen content in the transplanted CDC cells at approximately 20%. This ensured stem cell survival within the system, effectively enhancing diabetic foot wound healing. This outcome aligns with the three-week oxygen release kinetics observed across different microsphere content groups, corroborating the hypothesis that CDCs maintain cell survival by utilizing oxygen released by microspheres to ensure intracellular oxygen content. Upon injecting CDCs into the transplantation system, the favorable engraftment survival rate suggests that the oxygen-releasing microspheres in this study can continuously supply oxygen within the environment, ensuring stem cell viability. Consequently, this leads to a better outcome in promoting wound healing in diabetic foot ulcers of the animal model. Both in vitro and in vivo results support the system’s positive impact on improving diabetic foot stem cell therapies. However, the three-week duration may not suffice for complete tissue repair, such as vascularization and re-epithelialization, necessitating a longer oxygen release period to ensure stem cell functionality. Consequently, optimizing microsphere materials to extend oxygen release performance will be the focus of our future research.

Stem cell therapy mechanisms are linked to paracrine actions. Stem cells accelerate wound healing through paracrine pathways, enhancing epithelialization, granulation tissue formation, and angiogenesis for damaged tissue repair [44]. Angiogenesis poses a significant challenge to diabetic foot wound healing. A study by Lucíola et al. discovered that human CD133 stem cells stimulate angiogenesis and activate Wnt signaling through paracrine action, promoting diabetic ischemic ulcer healing [45]. In Lei et al. study [46], neurotrophic factor-3 (NT-3), which promotes nerve and vascular regeneration, accelerated diabetic foot wound healing by enhancing MSC paracrine effects. These findings suggest that stem cell therapy primarily accelerates vascular regeneration in diabetic foot conditions mainly by promoting paracrine effects, thus expediting wound healing—similar to our study. Our research showed that growth factors like bFGF, HGF, and VEGF, which encourage vascularization and cell survival, were highly expressed in aerobic environments compared to hypoxic conditions. This implies that the aerobic microenvironment provided by oxygen-releasing microspheres fosters stem cell paracrine effects, promoting not only their survival and growth but also re-epithelialization and wound healing of ulcerated wounds in animal models. Animal experiment results confirmed this observation. Another study reported that adipose-derived mesenchymal stem cells (ASCs) partially contribute to diabetic foot skin wound healing by increasing new blood vessels and collagen synthesis at the wound site [47]. In our research, ulcer repair in diabetic foot model mice was also achieved through the aforementioned mechanism, resulting in damaged skin contraction and healing. The upregulated mRNA expression levels of α-SMA, Collagen I, CD31, and others support this perspective. Simultaneously, the experiment determined CDCs improvement of diabetic foot animal model ulcer wounds through specific indicators. Further experimental analysis and verification are needed to ascertain whether the stem cell transplantation system developed in this study demonstrates superior therapeutic effects on other stem cell transplantations.

This study explores an oxygen-releasing stem cell transplantation system based on nanoscale hydrogel technology, aimed at promoting wound healing by maintaining intracellular oxygen levels, with potential applications in treating diabetic foot ulcers. However, clinical treatment of diabetic foot ulcers is not limited to wound healing but also involves blood sugar control and infection prevention. The limitation of this research lies in its focus solely on promoting wound healing, without considering the multifaceted factors in diabetic foot ulcer treatment. Moreover, the technology has not yet been validated in clinical practice, so its efficacy and safety still require further assessment. Lastly, this technology addresses wound healing exclusively, without effective methods for etiological treatment and prevention of diabetic foot ulcers. Future research directions should address diabetic foot ulcer treatment from multiple perspectives, including blood sugar control, infection prevention, and etiological treatment, integrating various therapeutic approaches to enhance outcomes. Additionally, more advanced nanotechnology and stem cell therapy techniques can be explored, offering more comprehensive and effective solutions for diabetic foot ulcer treatment.

## Conclusion

In this study, we developed a stem cell transplantation system utilizing a novel hydrogel and oxygen-releasing microspheres, achieving oxygen-release performance for over three weeks. This transplantation system significantly enhances stem cell retention and survival rates in diabetic foot target tissues. Through the paracrine pathway, the system suppresses the expression of inflammatory factors such as IL-6, promotes the upregulation of associated growth factors, and improves ulcer tissue vascularization in murine dorsal regions, thereby accelerating wound healing. This approach offers a promising strategy for the clinical implementation of localized stem cell delivery to improve diabetic foot wound healing.

## Data Availability

The datasets used and/or analysed during the current study are available from the corresponding author on reasonable request.

## References

[1] Boulton AJ, Vileikyte L, Ragnarson-Tennvall G, Apelqvist J (2005). The global burden of diabetic foot disease. The Lancet.

[2] Fu XL, Ding H, Miao WW, Mao CX, Zhan MQ, Chen HL (2019). Global recurrence rates in diabetic foot ulcers: a systematic review and meta-analysis. Diabetes Metab Res Rev.

[3] Jiacheng Z, Xinlong M, Jianxiong M, Hongqiang J, Pengfei L, Yanjun L, Fengbo L, Zhe H, Xuan J, Jingbo K. Epidemiological study on the incidence of deep vein thrombosis associated with fracture sites. 2016.

[4] Guo J, Dardik A, Fang K, Huang R, Gu Y (2017). Meta-analysis on the treatment of diabetic foot ulcers with autologous stem cells. Stem Cell Res Ther.

[5] Kirana S, Stratmann B, Lammers D, Negrean M, Stirban A, Minartz P, Koerperich H, Gastens M, Götting C, Prohaska W (2007). Wound therapy with autologous bone marrow stem cells in diabetic patients with ischaemia-induced tissue ulcers affecting the lower limbs. Int J Clin Pract.

[6] Shi R, Lian W, Jin Y, Cao C, Han S, Yang X, Zhao S, Li M, Zhao H (2020). Role and effect of vein-transplanted human umbilical cord mesenchymal stem cells in the repair of diabetic foot ulcers in rats. Acta Biochim Biophys Sin (Shanghai).

[7] Moon KC, Chung HY, Han SK, Jeong SH, Dhong ES (2019). Possibility of injecting adipose-derived stromal vascular fraction cells to accelerate Microcirculation in Ischemic Diabetic feet: a pilot study. Int J Stem Cells.

[8] Gorecka J, Kostiuk V, Fereydooni A, Gonzalez L, Luo J, Dash B, Isaji T, Ono S, Liu S, Lee SR, Xu J, Liu J, Taniguchi R, Yastula B, Hsia HC, Qyang Y, Dardik A (2019). The potential and limitations of induced pluripotent stem cells to achieve wound healing. Stem Cell Res Ther.

[9] Zhang Y, Zhao Y, Song B, Liu K, Gu J, Yue Y, Xiong R, Huang C (2021). UV-fluorescence probe for detection ni(2+) with colorimetric/spectral dual-mode analysis method and its practical application. Bioorg Chem.

[10] Zhang X, Qu Q, Yang A, Wang J, Cheng W, Deng Y, Zhou A, Lu T, Xiong R, Huang C (2023). Chitosan enhanced the stability and antibiofilm activity of self-propelled prussian blue micromotor. Carbohydr Polym.

[11] WeiWei H, Yi Y. Proceedings of the 2nd Summit Forum on Surgical Treatment of Rib Fractures and Thoracic Trauma Forum. Chinese Journal of Trauma. 2021;37(8):1.

[12] Feng W, Wang Z (2022). Shear-thinning and self-healing chitosan-graphene oxide hydrogel for hemostasis and wound healing. Carbohydr Polym.

[13] Divband B, Aghazadeh M, Al-Qaim ZH, Samiei M, Hussein FH, Shaabani A, Shahi S, Sedghi R (2021). Bioactive chitosan biguanidine-based injectable hydrogels as a novel BMP-2 and VEGF carrier for osteogenesis of dental pulp stem cells. Carbohydr Polym.

[14] Shi M, Gao Y, Lee L, Song T, Zhou J, Yan L, Li Y (2022). Adaptive gelatin Microspheres enhanced Stem Cell Delivery and Integration with Diabetic Wounds to activate skin tissue regeneration. Front Bioeng Biotechnol.

[15] Guan Y, Gao N, Niu H, Dang Y, Guan J (2021). Oxygen-release microspheres capable of releasing oxygen in response to environmental oxygen level to improve stem cell survival and tissue regeneration in ischemic hindlimbs. J Control Release.

[16] Wei ZZ, Zhu YB, Zhang JY, McCrary MR, Wang S, Zhang YB, Yu SP, Wei L (2017). Priming of the cells: hypoxic preconditioning for Stem Cell Therapy. Chin Med J (Engl).

[17] Thom SR, Bhopale VM, Velazquez OC, Goldstein LJ, Thom LH, Buerk DG. Stem cell mobilization by hyperbaric oxygen. American Journal of Physiology-Heart and Circulatory Physiology; 2006. 10.1152/ajpheart.00888.2005 16299259

[18] Oh SH, Ward CL, Atala A, Yoo JJ, Harrison BS (2009). Oxygen generating scaffolds for enhancing engineered tissue survival. Biomaterials.

[19] Ng S-M, Choi J-Y, Han H-S, Huh J-S, Lim JO (2010). Novel microencapsulation of potential drugs with low molecular weight and high hydrophilicity: hydrogen peroxide as a candidate compound. Int J Pharm.

[20] Chin K, Khattak SF, Bhatia SR, Roberts SC (2008). Hydrogel-perfluorocarbon composite scaffold promotes oxygen transport to immobilized cells. Biotechnol Prog.

[21] Bae SE, Son JS, Park K, Han DK (2009). Fabrication of covered porous PLGA microspheres using hydrogen peroxide for controlled drug delivery and regenerative medicine. J Controlled Release.

[22] Wen P, Zhu DH, Feng K, Liu FJ, Lou WY, Li N, Zong MH, Wu H (2016). Fabrication of electrospun polylactic acid nanofilm incorporating cinnamon essential oil/beta-cyclodextrin inclusion complex for antimicrobial packaging. Food Chem.

[23] Swaroop C, Shukla M (2018). Nano-magnesium oxide reinforced polylactic acid biofilms for food packaging applications. Int J Biol Macromol.

[24] Niu X, Liu Y, Song Y, Han J, Pan H (2018). Rosin modified cellulose nanofiber as a reinforcing and co-antimicrobial agents in polylactic acid /chitosan composite film for food packaging. Carbohydr Polym.

[25] Zong D, Zhang X, Yin X, Wang F, Yu J, Zhang S, Ding B (2022). Electrospun Fibrous Sponges: Principle, Fabrication, and applications. Adv Fiber Mater.

[26] Deng Y, Lu T, Zhang X, Zeng Z, Tao R, Qu Q, Zhang Y, Zhu M, Xiong R, Huang C (2022). Multi-hierarchical nanofiber membrane with typical curved-ribbon structure fabricated by green electrospinning for efficient, breathable and sustainable air filtration. J Membr Sci.

[27] Cao Y, Gang X, Sun C, Wang G (2017). Mesenchymal stem cells improve Healing of Diabetic Foot Ulcer. J Diabetes Res.

[28] Assi R, Foster TR, He H, Stamati K, Bai H, Huang Y, Hyder F, Rothman D, Shu C, Homer-Vanniasinkam S (2016). Delivery of mesenchymal stem cells in biomimetic engineered scaffolds promotes healing of diabetic ulcers. Regen Med.

[29] Niu H, Li X, Li H, Fan Z, Ma J, Guan J (2019). Thermosensitive, fast gelling, photoluminescent, highly flexible, and degradable hydrogels for stem cell delivery. Acta Biomater.

[30] Li Z, Wang F, Roy S, Sen CK, Guan J (2009). Injectable, highly flexible, and thermosensitive hydrogels capable of delivering superoxide dismutase. Biomacromolecules.

[31] Zheng Q, Li Z, Watanabe M (2022). Production of solid fuels by hydrothermal treatment of wastes of biomass, plastic, and biomass/plastic mixtures: a review. J Bioresources Bioprod.

[32] Luo J, Liu TL (2023). Electrochemical valorization of lignin: Status, challenges, and prospects. J Bioresources Bioprod.

[33] Sun P, Jiao J, Wang X, Chen L, Chen Z, Zhang K, Qu K, Qin X, Yang Z, Zhong JL, Wu W (2023). Nanomedicine hybrid and catechol functionalized chitosan as pH-responsive multi-function hydrogel to efficiently promote infection wound healing. Int J Biol Macromol.

[34] Zheng K, Tong Y, Zhang S, He R, Xiao L, Iqbal Z, Zhang Y, Gao J, Zhang L, Jiang L, Li Y (2021). Flexible bicolorimetric Polyacrylamide/Chitosan hydrogels for Smart Real-Time Monitoring and Promotion of Wound Healing. Adv Funct Mater.

[35] Makarevich PI, Boldyreva MA, Gluhanyuk EV, Efimenko AY, Dergilev KV, Shevchenko EK, Sharonov GV, Gallinger JO, Rodina PA, Sarkisyan SS, Hu YC, Parfyonova YV (2015). Enhanced angiogenesis in ischemic skeletal muscle after transplantation of cell sheets from baculovirus-transduced adipose-derived stromal cells expressing VEGF165. Stem Cell Res Ther.

[36] Niu H, Li C, Guan Y, Dang Y, Li X, Fan Z, Shen J, Ma L, Guan J (2020). High oxygen preservation hydrogels to augment cell survival under hypoxic condition. Acta Biomater.

[37] Li Z, Guo X, Guan J (2012). An oxygen release system to augment cardiac progenitor cell survival and differentiation under hypoxic condition. Biomaterials.

[38] Fan Z, Xu Z, Niu H, Gao N, Guan Y, Li C, Dang Y, Cui X, Liu XL, Duan Y, Li H, Zhou X, Lin PH, Ma J, Guan J (2018). An Injectable Oxygen Release System to Augment Cell Survival and promote Cardiac Repair following myocardial infarction. Sci Rep.

[39] Daly S, Thorpe M, Rockswold S, Hubbard M, Bergman T, Samadani U, Rockswold G (2018). Hyperbaric oxygen therapy in the treatment of acute severe traumatic brain Injury: a systematic review. J Neurotrauma.

[40] Coronel MM, Geusz R, Stabler CL (2017). Mitigating hypoxic stress on pancreatic islets via in situ oxygen generating biomaterial. Biomaterials.

[41] Sthijns M, van Blitterswijk CA, LaPointe VLS (2018). Redox regulation in regenerative medicine and tissue engineering: the paradox of oxygen. J Tissue Eng Regen Med.

[42] Maio A, Scaffaro R, Lentini L, Piccionello AP, Pibiri I (2018). Perfluorocarbons–graphene oxide nanoplatforms as biocompatible oxygen reservoirs. Chem Eng J.

[43] Kim HY, Kim SY, Lee HY, Lee JH, Rho GJ, Lee HJ, Lee HC, Byun JH, Oh SH (2019). Oxygen-releasing microparticles for cell survival and differentiation ability under Hypoxia for effective bone regeneration. Biomacromolecules.

[44] An T, Chen Y, Tu Y, Lin P (2021). Mesenchymal stromal cell-derived extracellular vesicles in the treatment of diabetic foot ulcers: application and challenges. Stem cell reviews and reports.

[45] Barcelos LS, Duplaa C, Krankel N, Graiani G, Invernici G, Katare R, Siragusa M, Meloni M, Campesi I, Monica M, Simm A, Campagnolo P, Mangialardi G, Stevanato L, Alessandri G, Emanueli C, Madeddu P (2009). Human CD133 + progenitor cells promote the healing of diabetic ischemic ulcers by paracrine stimulation of angiogenesis and activation of wnt signaling. Circ Res.

[46] Shen L, Zeng W, Wu YX, Hou CL, Chen W, Yang MC, Li L, Zhang YF, Zhu CH (2013). Neurotrophin-3 accelerates wound healing in diabetic mice by promoting a paracrine response in mesenchymal stem cells. Cell Transpl.

[47] Hu L, Wang J, Zhou X, Xiong Z, Zhao J, Yu R, Huang F, Zhang H, Chen L (2016). Exosomes derived from human adipose mensenchymal stem cells accelerates cutaneous wound healing via optimizing the characteristics of fibroblasts. Sci Rep.

